# Cost-effectiveness of treating multidrug-resistant tuberculosis in treatment initiative centers and treatment follow-up centers in Ethiopia

**DOI:** 10.1371/journal.pone.0235820

**Published:** 2020-07-27

**Authors:** Senait Alemayehu, Amanuel Yigezu, Damen Hailemariam, Alemayehu Hailu

**Affiliations:** 1 Ethiopian Public Health Institute, Addis Ababa, Ethiopia; 2 School of Public Health, Addis Ababa University, Addis Ababa, Ethiopia; 3 Department of Global Public Health and Primary Care, Bergen Center for Ethics and Priority Setting, University of Bergen, Bergen, Norway; London School of Hygiene and Tropical Medicine, UNITED KINGDOM

## Abstract

**Background:**

In Ethiopia, MDR-TB has become a significant public health threat; therefore, the Ministry of Health introduced two treatment approaches for MDR-TB cases: treatment initiative center (TIC) and treatment follow-up center (TFC). TIC is where patients usually are diagnosed and start the treatment. At TFC, we follow MDR-TB patients until they completed the treatment. However, there is no evidence about the cost-effectiveness of the approaches. Therefore, this study aimed to analyze the cost-effectiveness of MDR-TB treatment in TIC and TFC.

**Methods:**

In this study, we employed a full economic evaluation from a providers' perspective. We followed a hypothetical cohort of individuals from the age of 15 for a lifetime using a Markov model with five mutually exclusive health states. We used both primary and secondary data sources for the study. Ingredient-based costing approach was used. The costs include healthcare provider costs (recurrent and capital cost) and patient-side costs (direct and indirect). We use a human capital approach to estimate the indirect cost. The cost estimates were reported in the 2017 United States Dollar (US$), and effectiveness was measured using disability-adjusted life-years (DALYs) averted. Both costs and health benefits were discounted using a 3% discount rate. Both average and incremental cost-effectiveness ratios (ICER) were reported calculated. One-way and probabilistic sensitivity analyses were reported to determine the robustness of the estimates.

**Results:**

The cost per HIV negative patient successfully treated for MDR-TB was $8,416 at TIC and $6,657 at TFC. The average cost-effectiveness ratio per DALY averted at TFC was $671 and $1,417 per DALY averted at TIC. The incremental cost-effectiveness ratio (ICER) of MDR-TB treatment at TIC was $1,641 per DALYs averted.

**Conclusion:**

This study indicates that the treatment of MDR-TB at both TIC and TFC are cost-effective interventions compared with the willingness to pay threshold of three-times the GDP per capita in Ethiopia.

## Introduction

Multidrug-resistance Tuberculosis (MDR-TB) remains to be a considerable challenge globally and in Ethiopia. Ethiopia is one of the top 10 high MDR-TB burden countries, and studies indicate that about 1600 new MDR-TB cases were reported in 2018 [1, 2]. Treatment of patients with MDR-TB is more complicated than drug-susceptible cases because of reasons related to adherence, adverse effect of the drugs, and cost. World Health Organization (WHO) widely recommended that standard MDR-TB procedure should continue for a minimum of 20 months and at least 18 months after the patient becomes culture-negative. Chronic MDR-TB patients with extensive pulmonary disease may require treatment for 24 months or longer [1].

For a long time, MDR-TB treatment in Ethiopia has been typically delivered using the WHO DOTS-Plus model and involves prolonged inpatient treatment, more frequent monitoring of adverse drug reactions, ensures adherence, and may prevent spread within the community [3]. Recently, the Ethiopian Federal Ministry of Health (FMoH) developed a national MDR-TB treatment guideline, slightly modifying the recommendations outlined in the WHO guideline. This guide recommends two distinct approaches/level for MDR-TB treatment. The first is Treatment Initiation Centers (TIC), where patients usually are diagnosed and start the therapy. The second one is Treatment Follow-up Centers (TFC), where we follow the MDR-TB patients until they complete the MDR-TB treatment [4–6].

On the one hand, the TICs operate at the hospital level, and therefore, require designated space (i.e., inpatient ward and isolated outpatient spaces). The TICs are used for identifying MDR-TB cases, preparing the patients for the full course of the treatment, initiation of therapy with second-line drugs, and admitting severe cases and those with serious complications. On the other hand, the TFCs operate at the health center level, and they are responsible for managing all patients transferred from TIC. Besides, TFCs are accountable for the active finding of MDR-TB cases [1, 7]. Although there is a direct referral linkage between TIC and TFC, there is some evidence that both can separately provide MDR treatment.

There are a few studies on the cost-effectiveness of MDR-TB treatment approaches, and the results are mixed [8]. For instance, a study from India in 2017 estimated the total treatment cost for centralized MDR treatment (hospital-based model) to be about $3390 and US$ 1724 for the decentralized model. According to this study, the decentralized model can potentially save about $1666 per case, with an ICER of US$ 2383 per QALY gained. This study showed that the decentralized treatment of MDR-TB is cost-saving compared to centralized care [9]. Another study from Nigeria estimates that facility-based MDR treatment costs $2095 for facility-based care and US$ 1535 for home-based care, a potential saving of 25% [10].

The most common MDR-TB treatment modality in Ethiopia was that patients should be admitted for the full course of the treatment duration at TIC. Recently, the national MDR-TB treatment guideline recommended a new protocol that the intensive phase should be provided at TIC, and then patients should be linked to health centers (TFC) for completion of the remaining phase of the therapy. Furthermore, recent evidence suggests that health centers (TFC) can also deliver the full course of MDR-TB treatment as good as TIC (hospital). However, there is no evidence on the cost implication and cost-effectiveness of providing MDR treatment services through either TIC or TFC in Ethiopia. As a pilot, there are selected health centers in Addis Ababa, which started to provide the full course of MDR-TB treatment. Therefore, this study aimed to compare the cost-effectiveness of TIC and TFC as a fully-fledged MDR-TB treatment modality.

## Methods

### Study setting and sampling

We conducted this study in Addis Ababa, a capital city of Ethiopia, with more than 3.4 million population, about 100 health centers, and 11 hospitals. Both primary and secondary data sources were used to collect patient and provider costs and outcomes from a record of patient history. Taking in to account the MDR-TB caseload, we collected primary data from 2 hospitals (St. Peter specialized Hospital and ALERT Hospitals serving as TICs), and 13 health centers serving as TFCs. We collected the data from January 01 to April 30, 2018. A two-year (March 2014—March 2016) retrospective review of treatment outcomes of patients’ medical records was collected for MDR-TB cases. A sample of 243 MDR-TB patients with ages higher than 15 years were enrolled.

### Costing approach

#### Identification of cost categories

We applied the WHO costing guidelines for tuberculosis interventions in this study [11]. A micro-costing approach was applied to estimate the cost-effectiveness of MDR-TB treatment at TIC and TFC from the healthcare provider's perspective. All cost items related to MDR-TB treatment were identified based on standard national TB treatment guidelines. The costs are classified into recurrent and capital costs. Recurrent costs were defined as costs used with a duration of less than a year, whereas capital costs were expected to last longer than a year [12]. The recurrent costs include personnel, supplies, and utilities. Laboratory technologists, pharmacists, nurses, health officers, and other administrative staff were accounted at both TIC and TFC. Besides, at TIC, additional internists, radiologists, physicians were accounted as personnel costs. The capital costs included were equipment and building. Building/ space used for laboratory, waiting room, outpatient departments (OPD), inpatient admission room, and other administration staff room were included.

In addition, patient side costs were also collected from 243 MDR-TB patients enrolled in the study. From the patient side, we took in to account direct medical costs (consultations, admissions, laboratory investigations, and drugs), direct non-medical costs (transportation, food, healthcare visits, and lodging) and indirect costs (lost productivity due to the illness).

### Measurement of costs

Price data for most of the items were directly taken from the health center and hospital finance offices. MDR-TB drug cost, investigation, fuel, reagent, and other supply costs were collected from an interview with the TB centers team heads.

Besides, measurement of direct medical, direct non-medical, and indirect costs were measured by preparing a standard questionnaire and asking those patients with a history of MDR-TB in this study.

### Valuation of costs

Recurrent cost items such as drug, investigation, reagent, and supply cost were estimated by the average unit cost per patient multiplied by the number/amount used per TSR. Capital costs were annuitized based on their useful life-years, initial unit price, and consumer price index to account for annual inflation. The number of clients served was used as an allocation base to distribute the shared cost categories. Patients’ indirect (productivity loss) costs were estimated based on the human capital approach (HCA) [13].

Data entry and analysis were conducted with Microsoft excel 2016 [14], and the various costs collected in Ethiopian Birr were converted into United States Dollars (USD) at an official exchange rate of the National Bank of Ethiopia of the year 2017 (1USD = 23.55 ETB). All the costs were adjusted for inflation using a consumer price index of the year 2017 as a base year cost. Finally, we reported all costs in 2017 US Dollars.

### Measurement of health effect

The health outcome of the interventions was evaluated in terms of disability-adjusted life years (DALY) averted. The DALYs were calculated by adding years of life lost (YLL) and years of life lived with disability (YLD) [15]. Disability weights information to estimate the DALY was retrieved from the 2013 Global Burden of Disease study [16], and the WHO life tables were used to account for death from all other causes [5]. TB mortality rate of 26 per 100000 untreated cases was used [17]. The inputs used to estimate the health effect are presented in Table 1.

**Table 1:** Probabilities and costs (2017 USD) used in the cost-effectiveness analysis model.

Model inputs	Base-value	Minimum	Maximum	SD	Distribution	Source
Probability of healthy person getting TB infection	0.00182	0.00199	0.00353	0.00039	Beta	[22]
TB treatment coverage in Ethiopia	0.69	0.51	0.98	0.12	Beta	[22]
DS TB Treatment success at TFC	0.76	0.78	92.00	22.81	Beta	[6, 21]
DS TB failure at TFC	0.01	0.00	0.01	0.00	Beta	[6, 21]
DS TB death at TFC	0.05	0.02	0.06	0.01	Beta	[21, 23]
DS TB Treatment success at TIC	0.84	0.78	0.93	0.04	Beta	[21]
DS TB failure at TIC	0.01	0.00	0.03	0.01	Beta	[6, 21]
DS TB death at TIC	0.07	0.04	0.30	0.07	Beta	[6, 21]
Probability of healthy person getting MDR-TB	0.000027	0.000015	0.00004	0.000006	Beta	[22]
Probability of previously treated TB patient getting MDR-TB	0.000140	0.000036	0.00025	0.000054	Beta	[22]
MDR-TB failure at TIC	0.02	0.012	0.048	0.010	Beta	Primary data
MDR-TB death at TIC	0.013	0.093	0.30	0.05	Beta	Primary data
MDR-TB treatment success at TIC	0.598	0.54	0.8	0.07	Beta	Primary data
MDR-TB defaulter at TIC	0.26	0.1	0.34	0.06	Beta	Primary data
MDR-TB death at TFC	0.09	0.049	0.19	0.04	Beta	Primary data
MDR-TB treatment success at TFC	0.796	0.74	0.93	0.05	Beta	Primary data
MDR-TB default rate at TFC	0.16	0.1	0.22	0.03	Beta	Primary data
Cost of DS TB at TIC (USD)	260	160	500	85	Gama	[20, 24]
Cost of DS TB at TFC (USD)	162	160	260	25	Gama	[20, 24]
Cost of MDR-TB HIV negative pts at TIC (USD)	4,637	3,710	5,563	463	Gama	Primary data
Cost of MDR-TB HIV negative pts at TFC (USD)	3,328	2,458	3,668	302	Gama	Primary data
Disability weight of TB/MDR-TB	0.33	0.27	0.55	0.07	Beta	[20, 24, 25]
Probability of starting MDR-TB treatment	0.24	0.22	0.28	0.02	Beta	[22]
Discount rate (costs and effectiveness)	0.03	0.01	0.06	-	Beta	[12]

### Cost-Effectiveness Analysis (CEA)

A full economic evaluation was conducted to compare the cost-effectiveness of the MDR-TB treatment (i.e., TIC and TFC) in a hypothetical cohort of MDR-TB patients from a provider's perspective. Markov state transition model was developed using TreeAge Pro Suit 2018 (© 2018 TreeAge Software, Inc.). Both costs and health outcomes were discounted with a 3% discount rate and reported in the 2017 US Dollar (US$). Average cost-effectiveness ratio (ACER) and an incremental cost-effectiveness ratio (ICER) were reported comparing the strategy with a ‘do-nothing’ option. The ICER and ACER were reported in cost per DALYs averted [18]. The WHO-CHOICE framework was applied to define decision and to make a judgment that the interventions are cost-effective or not. An intervention that costs less than three times the national GDP per capita per DALY averted was considered as ‘cost-effective.' An intervention that costs less than one times the national GDP per capita was considered as ‘very cost-effective’ intervention. If the intervention costs more than three times, the national GDP per capita was considered as ‘not cost-effective' [13].

### Model structure of the CEA

**Fig 1:**
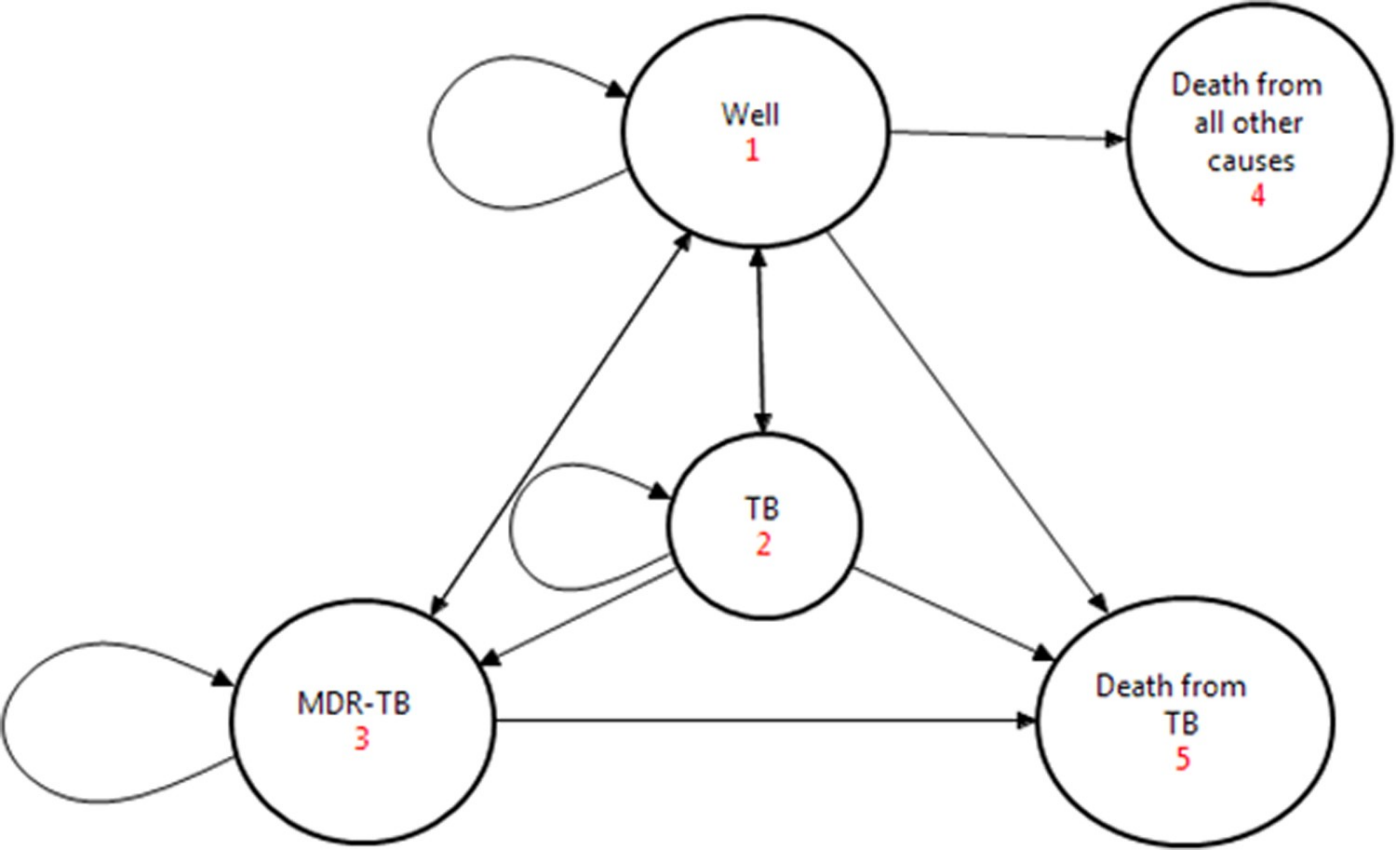
Markov tree state-transitions diagram.

Markov model different transition states to analyze the long-term outcomes, costs, and effects that were spread over a long time [19]. The model was structured in five health states: Well, TB, MDR-TB, death from TB, and death from other causes. Patients were modeled to start from a ‘well’ state. In any given time, the individual would be only in one health state (Fig 1). The transition probabilities are the probabilities in which a subject moves from one state to another within a given cycle-length. The starting age for the simulated model was fifteen years with a lifetime horizon and a cycle length of one-year. The transition probability data is presented in Table 1.

We took a cure rate of about 70% of tuberculosis cases (TSR) [20, 21], while some might not be diagnosed and remain with the disease. When adequately treated, death from tuberculosis would be infrequent; therefore, we assume zero mortality while improper treatment of cases could progress to MDR-TB. Therefore, we took a mortality assumption of 26 per 100,000 untreated cases [20]. Besides, we assumed all patients who were treated for MDR-TB complete their treatment at the facility they first start it.

### Sensitivity analysis

Both one-way and probabilistic sensitivity analysis (PSA) was conducted to check for the robustness of the finding by varying different inputs from the mean value.

The PSA conducted through Monte Carlo simulation is a form of statistical analysis in which the distribution of the use of variables uses a gamma distribution for the costs and a beta distribution for a health outcome. We assumed health outcomes variables to vary by 5% and costs by 20% from the baseline value. This assumption is used because some data related to costs were not easy to get, but there was a good record of the health outcomes of MDR-TB patients. The input parameters for the cost-effectiveness analysis are presented in Table 1.

### Ethics approval and consent to participate

The study was approved by the Institutional Review Board (IRB) of the College of Health Sciences at Addis Ababa University. Participation in the study was only voluntary, and the data were collected after informed written consent was obtained from all participants. Parental consent was obtained for those between the age of 15 and 18 years old.

## Results

Out of the 243 patients enrolled in the study, 130 (54%) were MDR-TB patients from new cases, while the rest 113 (46%) were MDR-TB patients from the previously treated cases. The majority of the study participants were male (52%), have educational status below the secondary level (78%) and in the age range of 15–34 (72%). About 70% of the study participants live in households with a family size of 3–6 people. The socio-demographic characteristics of the study participants are presented in Table 2.

**Table 2:** Study participants demographic characteristics.

Demographic variables	TIC	TFC	Overall
	n (%).	n (%).	n (%).
Sex			
Male	77 (60)	49 (43)	126 (52)
Female	51 (40)	66 ‘(57)	117 (48)
Age			
15–24	38 (30)	35 (30)	73 (30)
25–34	54 (41)	48 (42)	102 (42)
35–44	19 (15)	17 (15)	36 (15)
45–54	14 (11)	11 (9)	25 (10)
55–64	2 (2)	1 (1)	3 (1)
> 65	1 (1)	3 (3)	4 (2)
Marital status			
Married	48 (38)	66 (57)	114 (47)
Single	76 (59)	47 (41)	123 (51)
Divorced	4 (3)	2 (2)	6 (2)
Educational status			
Illiterate	13 (10)	15 (13)	28 (12)
Read and write	51 (40)	18 (16)	69 (28)
Primary education	51 (40)	42 (36)	93 (38)
Secondary education	10 (8)	31 (27)	41 (17)
College and Above	3 (2)	9 (8)	12 (5)
Occupation			
Farmer	2 (2)	0 (0)	2 (1)
Government employee	15 (12)	22 (19)	37 (15)
Self-employed	78 (60)	51 (44)	129 (53)
School student	8 (6)	14 (12)	22 (9)
Day laborer	9 (7)	8 (7)	17 (7)
Unemployed	15 (12)	20 (18)	35 (14)
Retired	1 (1)	0 (0)	1 (1)
Family size			
< 3	33 (26)	30 (26)	63 (26)
3–6	89 (69)	76 (66)	165 (68)
> 6	6 (5)	9 (8)	15 (6)
Overall	128 (100)	115 (100)	243 (100)

### MDR-TB treatment costs

On average, about 7 months were spent during MDR-TB treatment at both TIC and TFC, with an average of 80 days of admission. The mean treatment duration in the intensive and continuation phase was 8 and 10 months, respectively (Table 3).

**Table 3:** Intensive and continuation phase of patient costs, in 2017 USD.

*Cost Centers*	TIC			TFC		
	Intensive	Continuation	Total	Intensive	Continuation	Total
*HIV Positive*						
Transport Dot	389	-	389	253	-	253
Food Dot	320	-	320	209	-	209
Accommodation	-	52	52	-	-	0
Transport for follow up	-	41	41	-	64.87	64
Food for follow up	-	123	123	-	98	98
Indirect cost	996	-	996	818	-	818
Total Cost	1,706	216	1,922	1,280	162	1,443
*HIV Negative*						
Transport Dot cost	335	-	335.4	234	-	234
Food Dot cost	270	-	270.5	196	-	196
Accommodation cost	-	52	52.2	-	-	-
Transport for follow up	-	41	41.0	-	64.9	65
Food for follow up	-	123	123	-	98	98
Indirect cost	976	-	976.3	798	-	798
Total Cost	1,582	216	1,798	1,228	162.9	1,391

TIC: treatment initiation center; TFC: treatment follow up center.

Patient costs were all the same except for the admission cost, which was $ 946, which was 9% of the total HIV positive costs at TIC. The total cost for MDR-TB treatment for the HIV negative patients was $ 8,413 and $ 6,657 at TIC and TFC, respectively. The majority of the costs were associated with the variable cost of intensive care, about 70% for TIC, and 63% for TFC (Table 4).

**Table 4:** MDR-TB treatment costs per TSR at TIC and TFC, in 2017 USD.

	TIC				TFC			
Admit and HIV status	Average	Min	Max	SD	Average	Min	Max	SD
Admitted (HIV Positive)	11,979	9,583	14,375	3,388	9,250	7,400	11,100	2616
Not Admitted (HIV Positive)	9,614	7,691	11,537	2,719	7,859	6,287	9,431	2,222
Admitted (HIV Negative)	10,781	8,625	12,937	3,049	8,048	6,031	9,046	2,132
Not Admitted (HIV Negative)	8,416	7,420	11,130	2,623	6,657	4,918	7,377	1,738

TIC: treatment initiation center; TFC: treatment follow up center; Min: minimum; Max: maximum; SD: Standard deviation.

### Cost-effectiveness analysis

Table 5 presents the cost-effectiveness ratio of the treatment options. TFC strategy would require an average cost of $ 671 per DALY averted. The average cost-effectiveness ratio of TIC was $ 1,417 per DALY averted. The incremental cost of TIC to avert one more DALY was $ 1,641 on the costs of TFC.

**Table 5:** Cost, effectiveness, average and incremental cost-effectiveness ratio per DALYs averted.

Strategies	Cost (USD)	Incremental Cost	Eff. (DALYs)	Incremental Eff	ACER	ICER
TFC	15.448	-	0.023	-	671	-
TIC	24.096	8.648	0.017	0.005	1417	1641

### Probabilistic sensitivity analysis

**Fig 2:**
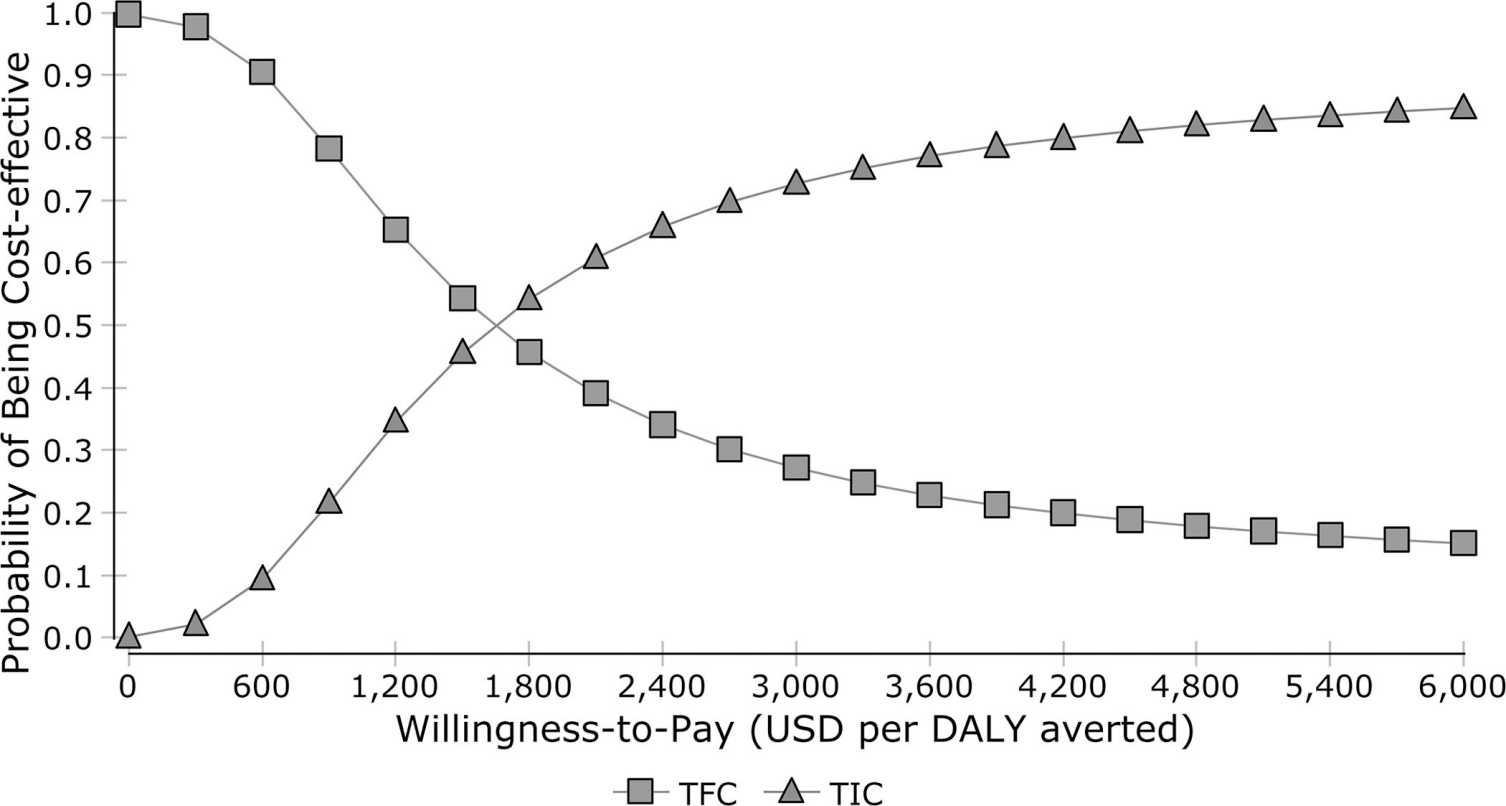
Cost-effectiveness acceptability curve.

MDR-TB treatment at TFC has an 80% probability of being very cost-effective compared to GDP threshold value; this was 20% for TIC. When compared with the three times GDP per capita threshold, TIC has a 65% probability of being cost-effective (Fig 2).

**Fig 3:**
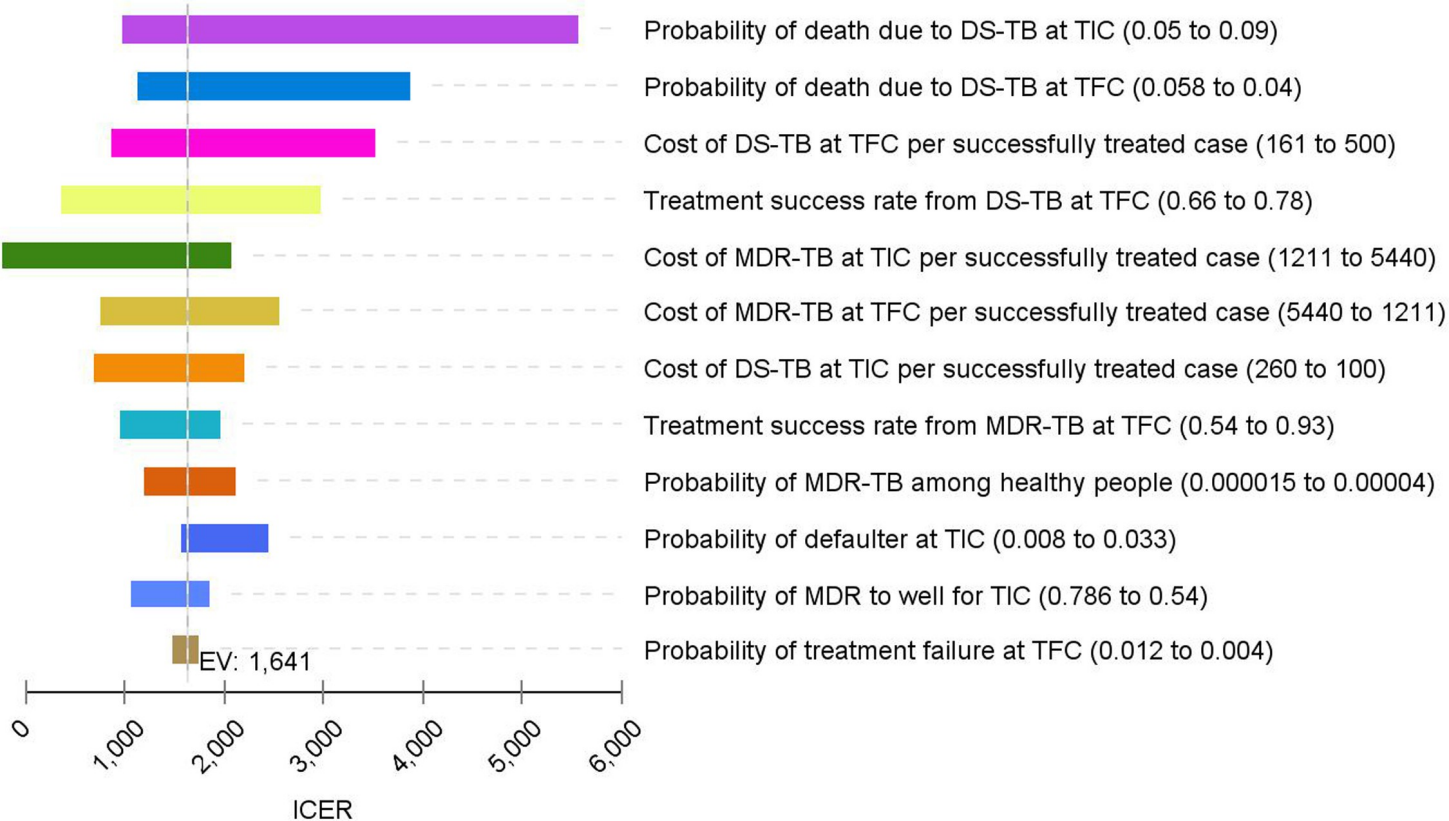
Tornado diagram—ICER per DALYs averted TIC vs. TFC.

**Fig 4:**
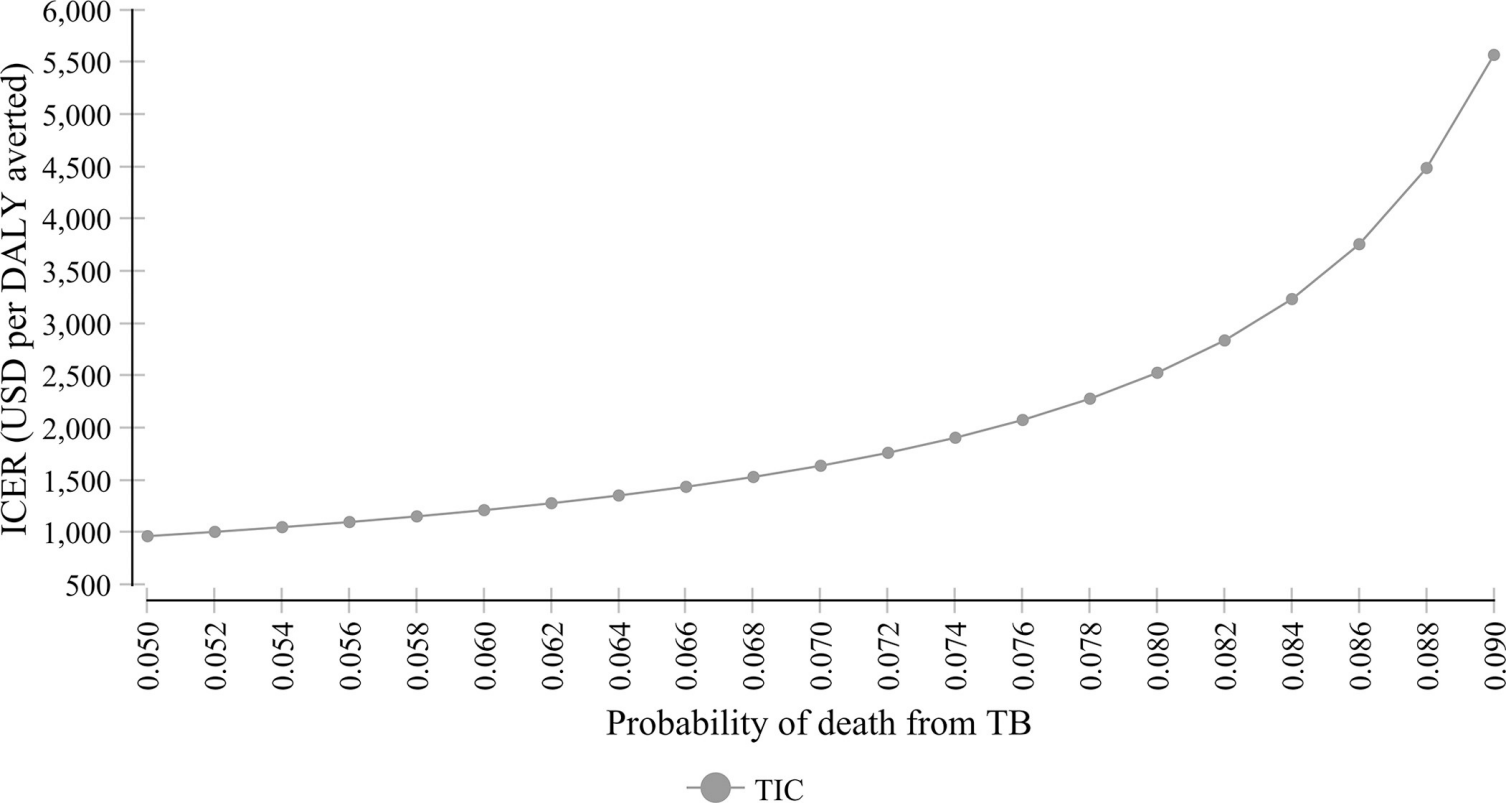
Sensitivity of ICER to variations in the probability of death from TB at hospitals.

The one-way sensitivity analysis using the tornado diagram (Fig 3), in general, indicates that the base case ICER finding was robust with a change in most of the parameters with expected minimum and maximum values. However, variation in the probability of death due to TB at hospitals substantially affects the ICER. The lower the probability of death at the hospital, the lower the ICER. The probability of death at the health center and cost of TB at a hospital also affect the base-case ICER substantially (Fig 4).

## Discussion

Therefore, this study can inform a policy choice in scaling up MDR-TB treatment in the Ethiopian essential health service package, along with a further budget impact analysis and affordability studies. In summary, our analysis provides evidence that the treatment of MDR-TB, both at TIC and TFC, was cost-effective options in Ethiopia based on the cost-effectiveness threshold of 3 times GDP per capita.

### Cost of MDR-TB treatment at TIC

MDR-TB treatment cost to patients is higher for TIC than TFC, especially during the intensive phase. This might be related costs associated with accommodation, transportation, and food due to the long distance to reach the TIC centers. The costs of TIC in our study is lower than studies from South Africa and higher than studies from Brazil, Bangladesh, and Tanzania. The difference in cost might be due to variation in the country’s economic status, the implemented model of care, the cost component included in the studies such as indirect costs, and the model used to estimate the costs [4].

The cost of providing the service at TIC is more costly than TFC because of the higher personnel and equipment cost TIC. The mean cost for MDR-TB treatment per patient at TIC was $8416.17 for HIV negative non-admitted patients and 12% higher if the patient was admitted. The cost would increase by 10% if the patient were HIV positive because of additional investigation and drug cost. This finding is comparable with a find from studies conducted in Estonia ($8,974 in 2003 USD) and Tomsk Oblast ($10,088 in 2003 USD) [26]. However, the cost result we found in this study is lower than a finding from studies done in South Africa and Brazil; and higher than finding from Bangladesh and Tanzania [27].

### Cost of MDR-TB treatment at TFC

According to our study, the provider and patient costs of treating MDR-TB at TFC was 21% lower than TIC. We also found that MDR-TB drug cost and investigation costs are almost similar at both TIC and TFC. However, these cost estimates from other studies in South Africa, Brazil, Bangladesh, and Tanzania [5, 28] reporter higher compared with our estimate. However, the MDR-TB treatment per patient cost at the TIC and TFC in our study was yet is less than the WHO estimate of the MDR-TB cost per patient of $10,000 for Ethiopia in 2015 [20]. This result might be related to the omission of some costs like laboratory sample transport cost, culture media, personal and building cost from TIC, and TFC.

From the patients' perspective, follow-up transport cost is higher at TFC than TIC because MDR-TB patients at TFC are referred to TIC for sputum culture, other investigation, and physician consultation. The TFCs are usually located close by the patients' residence than the TICs, and therefore, the cost of transportation and lodging would be relatively lower at TFCs than at TICs.

### Cost-effectiveness of MDR-TB treatment at TIC and TFC

The ACER of TFC (health center) is very cost-effective when compared to ‘doing nothing’ option. The ACER of TIC also indicates that it is cost-effective. Although the MDR-TB treatment guideline operates through referral linkage between the two strategies, implementing both TIC and TFC as separate stand-alone operating centers can be cost-effective. Based on CET of 3 times GDP per capita for Ethiopia, this study indicated that MDR-TB treatment is cost-effective at TIC with an ICER of $1641 per DALY averted when compared to TFC. This result is comparable to a model-based analysis in Bangladesh (ICER = $1472 in 2017 USD). However, the ICER in our finding is somehow higher than the findings from Estonia ($773 in 2017 US$) and Tomsk ($573 in 2017 US$) [26].

This study is the first of its kind in Ethiopia that compared the cost and cost-effectiveness of MDR-TB treatment options. Primary patient-level data on cost and effectiveness of the interventions was collected and applied. However, some caveats deserve due consideration concerning the data and generalizability. For example, some direct and indirect costs of the caregivers and training costs were not included due to the data limitation. Similarly, patient-side costs before and after MDR-TB treatment were not accounted for in this study. This might slightly underestimate the actual unit cost of MDR-TB treatment. Besides, we were not able to get the price data for some of the items consumed in delivering the MDR treatment service, and therefore, the price information of similar items was taken from the market price.

## Conclusion

In conclusion, the economic cost of MDR-TB treatment was potentially high both to the patients and to health service providers. The ACER of TFC was $672 per DALY averted and $1,417 per DALY averted for TIC. The TIC has an ICER of $641 per DALY averted when compared to TFC, which is a cost-effective strategy since the ICER is less than three times GDP per capita per DALYs averted for Ethiopia.

## Supporting information

S1 File MDR TB costing. (XLSX)
